# Home and Online Management and Evaluation of Blood Pressure (HOME BP) digital intervention for self-management of uncontrolled, essential hypertension: a protocol for the randomised controlled HOME BP trial

**DOI:** 10.1136/bmjopen-2016-012684

**Published:** 2016-11-07

**Authors:** Rebecca Band, Katherine Morton, Beth Stuart, James Raftery, Katherine Bradbury, Guiqing Lily Yao, Shihua Zhu, Paul Little, Lucy Yardley, Richard J McManus

**Affiliations:** 1 Academic Unit of Psychology, University of Southampton, Southampton, UK; 2 Primary Care and Population Sciences, Faculty of Medicine, University of Southampton, Southampton, UK; 3 Faculty of Medicine, University of Southampton, Southampton, UK; 4 Nuffield Department of Primary Care Health Sciences, University of Oxford, Oxford, UK

**Keywords:** PRIMARY CARE

## Abstract

**Introduction:**

Self-management of hypertension, including self-monitoring and antihypertensive medication titration, lowers blood pressure (BP) at 1 year compared to usual care. The aim of the current trial is to assess the effectiveness of the Home and Online Management and Evaluation of Blood Pressure (HOME BP) intervention for the self-management of hypertension in primary care.

**Methods and analysis:**

The HOME BP trial will be a randomised controlled trial comparing BP self-management—consisting of the HOME BP online digital intervention with self-monitoring, lifestyle advice and antihypertensive drug titration—with usual care for people with uncontrolled essential hypertension. Eligible patients will be recruited from primary care and randomised to usual care or to self-management using HOME BP. The primary outcome will be the difference in mean systolic BP (mm Hg) at 12-month follow-up between the intervention and control groups adjusting for baseline BP and covariates. Secondary outcomes (also adjusted for baseline and covariates where appropriate) will be differences in mean BP at 6 months and diastolic BP at 12 months; patient enablement; quality of life, and economic analyses including all key resources associated with the intervention and related services, adopting a broad societal perspective to include NHS, social care and patient costs, considered within trial and modelled with a lifetime horizon. Medication beliefs, adherence and changes; self-efficacy; perceived side effects and lifestyle changes will be measured for process analyses. Qualitative analyses will explore patient and healthcare professional experiences of HOME BP to gain insights into the factors affecting acceptability, feasibility and adherence.

**Ethics and dissemination:**

This study has received NHS ethical approval (REC reference 15/SC/0082). The findings from HOME BP will be disseminated widely through peer-reviewed publications, scientific conferences and workshops. If successful, HOME BP will be directly applicable to UK primary care management of hypertension.

**Trial registration number:**

ISRCTN13790648; pre-results.

**Strengths and limitations of this study**

- The study develops existing efficacious antihypertensive titration procedures into an online format.
- Home and Online Management and Evaluation of Blood Pressure (HOME BP) combines medication intensification with lifestyle change and behavioural support.
- The study has been designed to facilitate implementation within a primary care context.

## Introduction

Blood pressure (BP) is a key risk factor for cardiovascular disease, the largest cause of morbidity and mortality worldwide.^1^ The health survey for England (2012) identified that ∼31% of men and 27% of women in England have hypertension,^2^ yet over one-third of all treated patients currently fail to reach national guidelines for BP control.^3^ The mean BP in the UK currently ranks among the highest in Europe (27th/35), and the benefits of a population-wide reduction to levels seen in countries such as Denmark and Spain (which are among the lowest) would mean a 10 mm Hg systolic blood pressure (SBP) reduction.^4^ A 10 mm Hg reduction in BP is estimated to lead to a 41% reduction in stroke and a 22% reduction in coronary heart disease (CHD).^5^ New strokes affect over 120 000 patients per annum in the UK, and stroke is the third leading cause of death and the most important cause of adult disability, representing 4.4% of direct National Health Service (NHS) costs (notwithstanding costs to social services, patients and carers).

Factors responsible for suboptimal BP control include those due to patients, physicians and the health system.^6^ The key patient factors are adherence to medication and other health behaviours.^7^ ^8^ Clinical inertia is another key issue, whereby clinicians fail to intensify treatment, despite evidence of inadequate control. It is estimated that clinical inertia may account for almost 20% of the variance in BP control,^9^ with evidence suggesting that treatment intensification fails to occur in nearly half (45%) of consultations in which patients had a single BP reading above target, and around a third (36%) with two successive readings above target.^10^ Lack of intensification may be due, in part, to the ‘white-coat effect’, where BP readings in clinic consultations are elevated in comparison to self-monitored home BP readings;^11^ ^12^ awareness that clinic readings may be inaccurate may contribute to clinician and patient reluctance to implement medication changes. Even with self-monitoring of BP, clinical inertia can be profound.^13^

There is evidence that self-monitoring BP at home is useful in improving medication adherence, reducing therapeutic inertia and controlling BP,^12^ ^14–17^ and research by our team and others has shown that sustained reductions in BP can indeed be achieved by linking self-monitoring to preplanned medication titration when hypertension is uncontrolled.^17–21^ It can also detect and treat masked uncontrolled hypertension.^22^ While some evidence suggests that aggressive titration of antihypertensive medication without behavioural support is optimal for controlling BP,^23^ further evidence suggests that greater reductions in BP may be achieved by the addition of behavioural support for self-monitoring.^19^ ^24^ ^25^ This may be further facilitated when combined with efficacious lifestyle changes, such as weight reduction, sodium restriction and increasing physical activity,^26^ ^27^ therefore HOME BP will combine all of these components.

### Rationale and risk-benefits for the current trial

Increasingly widespread access to the internet and mobile phones^28^ ^29^ means that healthcare digital interventions (DIs) (also known as online interventions or computer-based health interventions within the literature) are accessible to the majority of patients and can be used to provide information and support at any time the patient needs it.^30^ DIs can empower patients by providing better access to personalised information and support for active involvement in treatment and self-management.^30^ A large meta-analysis found a small but significant positive effect of DIs on health-related behaviours,^31^ while a Cochrane review found evidence that computer-based health interventions for those with chronic health conditions significantly improved knowledge, health behaviours and clinical outcomes.^32^ DIs have the potential to make significant savings by automating routine aspects of patient education, monitoring and support, freeing up health professional resources for when patients most need them.^33^ There is accumulating evidence that DIs can deliver better and more efficient healthcare in the context of hypertension,^34^ ^35^ confirmed by a recent systematic review and meta-analysis undertaken by our team.^36^ Therefore, the HOME BP programme has been developed to incorporate previously identified effective intervention components (self-monitoring, preplanned medication titrations and behavioural support) delivered in an acceptable and feasible online format.

### Study aims and research questions

The primary aim of the HOME BP trial is to assess the feasibility, acceptability, effectiveness and cost-effectiveness of adding the HOME BP intervention (comprising the HOME BP online DI, self-monitoring, medication titration and lifestyle interventions with nurse support) into primary care for the self-management of hypertension in comparison to usual care.

Main research question:

- Does the HOME BP intervention to assist self-monitoring and self-management of uncontrolled hypertension result in greater control of SBP after 1 year?

Secondary research questions:

- Does the HOME BP intervention to assist self-monitoring and self-management of uncontrolled hypertension result in greater control of SBP and diastolic blood pressure (DBP) after 6 months and DBP after 1 year?
- Is the HOME BP intervention more cost-effective than usual care in managing poorly controlled hypertension in primary care?

Process analysis research questions:

- Which factors are related to patient engagement and adherence to HOME BP?
- Which factors are related to patient medication adherence and uptake of recommended medication titrations?
- Does the inclusion of lifestyle change choices and behavioural support result in engagement with lifestyle change?
- Is the HOME BP intervention acceptable to patients when integrated into routine practice?
- What are patient and healthcare practitioner views and experiences of the HOME BP intervention and its addition into primary care for the self-management of poorly controlled hypertension?

## Methods and analysis

### Study design and setting

The HOME BP intervention for the self-management of high BP consists of the integrated patient and healthcare practitioner HOME BP online DI, BP self-monitoring, health professional directed and supervised titration of antihypertensive medication and user-selected lifestyle modifications, for people with uncontrolled essential hypertension. The HOME BP study is a pragmatic, randomised controlled trial comparing the HOME BP intervention with optional nurse support to a control group receiving usual hypertension care within a UK primary care setting.

### Study participants

#### Identification, follow-up and non-participation

Eligible participants will be identified from electronic database searches of clinical systems of collaborating general practices. Records of potentially eligible patients will be further screened by general practitioners (GPs) to remove participants known to meet exclusion criteria. Remaining potentially eligible patients will be invited by letter from the practice to attend a baseline clinic to establish eligibility, take consent, collect baseline data and randomise consented patients. Individuals not wanting to take part will be provided with a form to return should they wish to decline to take part in the trial. This will ask for basic demographic information and their reasons for declining, including an option not to give a reason if they prefer not to. The practice nurse (or other practice member as delegated by the lead GP) will have the option to follow-up invited patients by telephone after 1 month without response. All patient identification prior to consent will be conducted independently of the research team to maintain confidentiality.

#### Eligibility criteria

Eligibility criteria will aim to capture adult patients with treated but uncontrolled hypertension managed in primary care who will be aged 18 or over, have a Read code for hypertension in the clinical record (confirming hypertension), a current prescription of antihypertensive medication and a mean BP reading (calculated from the second and third BP readings) of >140/90 mm Hg taken during a baseline clinic appointment. Eligible patients will also be required to have access to the internet and be able to comprehend the website.

#### Exclusion criteria

Exclusion criteria will include an inability to self-monitor (eg, diagnosis of dementia), a mean baseline BP reading of >180/110 mm Hg (stage 3 hypertension requiring more urgent intervention than would be available in the trial), hypertension not managed by family doctor (the trial facilitates primary care titration of medication), prescribed more than three antihypertensive medications (ie, resistant hypertension), chronic kidney disease (CKD) stage 4–5 or CKD stage 3 not managed by the GP (likely to be under specialist care and requiring different BP targets), terminal disease or other condition which in the opinion of the family doctor makes them inappropriate to take part, pregnant or breast feeding, a history of proteinuria (albumin creatinine Ratio (ACR) >30 mg/mmol) (different BP targets), postural hypotension (>20 mm Hg systolic drop after 1 min standing) (for whom intensification of BP medication may be inappropriate), atrial fibrillation (self-monitoring with oscillometric equipment not suitable), an acute cardiovascular event in the previous 3 months (BP and antihypertensive medication may not be stable) or a household member already enrolled on the study (to avoid bias). Participants who are involved in any other BP research will not be able to take part in the study.

#### Randomisation

Participants will be randomised in a 1:1 ratio to receive either usual care or the HOME BP intervention with optional nurse support using the HOME BP online system. Minimisation will be used taking into account participants' baseline SBP, age, diabetes status and practice. Patients are randomised to the optimal group 80% of the time. Any random numbers the minimisation routine might need will be computer generated, therefore bypassing study team involvement.

#### Participant flow through the study

At baseline, all patients will receive a consultation where informed consent will be taken, as well as clinical measures (confirmation of raised BP, height, weight) and medical history. Hypoglycaemic episodes in the last 6 months will be recorded for diabetic patients. BP will be measured after 5 min rest with a validated electronic automated sphygmomanometer (BP TRU BPM 200). Participants will be seated, and BP will be measured in their left arm unless there is a reason not to (principally lymphoedema or other medical condition). Six BP readings will be taken at intervals of 1 min, with the mean of the second and third readings used for the primary outcome. Patients will complete demographic measures and baseline assessments at home via HOME BP online, following which automatic allocation to intervention or usual care group will be triggered. Patients will be immediately notified of their allocation through HOME BP online, confirmed by email or letter. Practice staff will also be notified of patient group allocation by email. Participants will be informed that the research team will have access to their personal and study data to stay in touch with them throughout the study.

**Figure 1:**
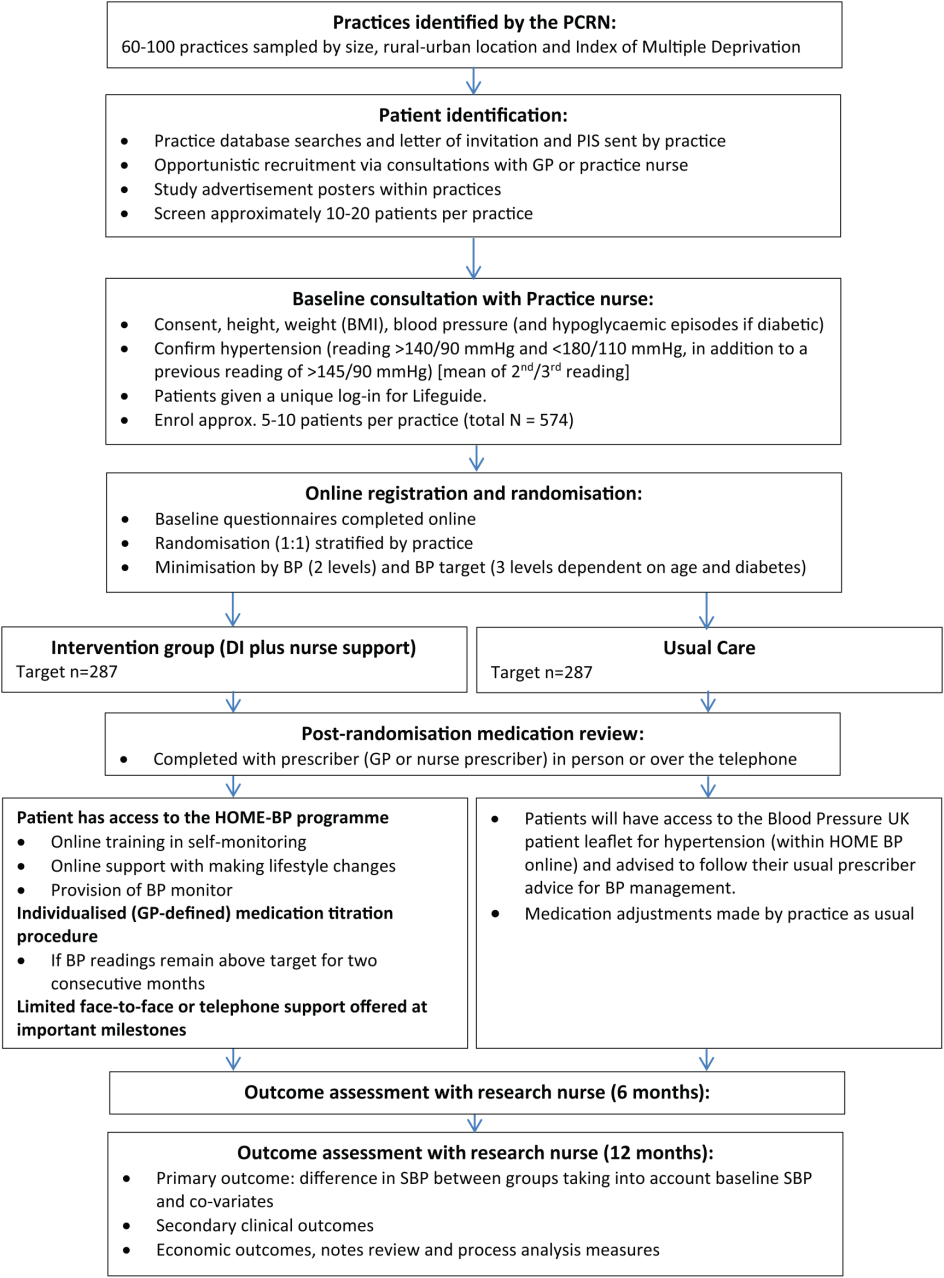
Participant flow through the HOME BP study. GPs, general practitioners; HOME BP, Home and Online Management and Evaluation of Blood Pressure.

Following randomisation, all patients will receive a BP medication review with a trained HOME BP prescriber (one or more GPs or nurse prescriber's nominated by the practice). For patients allocated to the intervention group, prescribers will be asked to select and agree an individualised medication titration plan (including three potential medication changes should BP remain above target). Participants allocated to usual care will receive a routine hypertension medication review. None of the study participants (patients or healthcare practitioners) will be blinded to group allocation. At 6-months and 12 months after randomisation, all patients will be invited to a follow-up appointment with an independent research nurse. This may take place within the clinic or at a participant's home. Patients will be asked to not reveal their group allocation at this appointment, as research nurses completing the 6-month and 12-month follow-up appointments will be blinded to the patient’s group. All follow-up BP measurements will be recorded no earlier than 2 weeks before the follow-up date, and no later than 4 weeks following the follow-up date at the 6-month follow-up. The follow-up period will be extended up to a maximum of 8 weeks for the 12-month follow-up. Patient BP readings and clinical measures will be taken in the same way as baseline and follow-up questionnaires completed through HOME BP online. Participant flow through the trial is outlined in figure 1. Participants will be withdrawn from the study if they become no longer eligible to participate (eg, due to pregnancy). Participants who choose not to continue with the study will be offered the opportunity to continue self-monitoring only rather than withdrawing completely from the study, and asked if they would be prepared to attend follow-up appointments.

#### Sample size consideration

A sample size of 244 patients per group is required in order to have 90% power to detect a difference in SBP of 5 mm Hg (SD 17 mm Hg) between intervention and control groups, based on the findings from the TASMINH-2 study.^20^ Allowing for a 15% participant drop out, we will seek to recruit 287 participants per condition, resulting in a total sample size of 574 participants.

#### Recruitment

On the basis of our pilot work, and previous experience from hypertension self-management interventions,^21^ we estimate an approximate recruitment rate of 4% from the practice invite mail-out. Assuming an average practice list size of 5000, with a prevalence of hypertension of 13%, it is estimated that ∼75% of the hypertension register will be excluded based on last BP reading and other practice exclusions. We estimate that ∼15% of patients invited will attend a baseline screening appointment, with ∼50% ineligible due to low BP readings during the screening appointment. We also estimate that a further 50% may decide not to participate due to the online format of the intervention. Around 95 practices are therefore likely to be required in order to recruit the target number of participants (n=574).

### HOME BP intervention

The HOME BP intervention consists of HOME BP online (for patients and healthcare professionals), BP self-monitoring, medication titration procedures and optional behavioural support. Each of these components is described below, guided by the TIDieR checklist where possible.^37^ The intervention was developed using a theory-based, evidence-based and person-based approach,^38^ which is fully described elsewhere (article in submission).

#### HOME BP online: patient behavioural components

HOME BP online will use behavioural techniques to build patient motivation for BP self-monitoring, medication adherence and medication titration (Band R, Bradbury K, Morton K, *et al*. Intervention planning for a digital intervention for self-management of hypertension: a theory-, evidence- and person-based approach. *Implement Sci*. In submission). This will include presenting the health-related benefits of self-monitoring and reducing BP through medication titration procedures. Positive patient outcome expectancies will be encouraged by outlining ways that using the HOME BP intervention may reduce the chances of experiencing negative health consequences associated with hypertension. Common patient concerns regarding medication side effects are also addressed.

**Figure 2:**
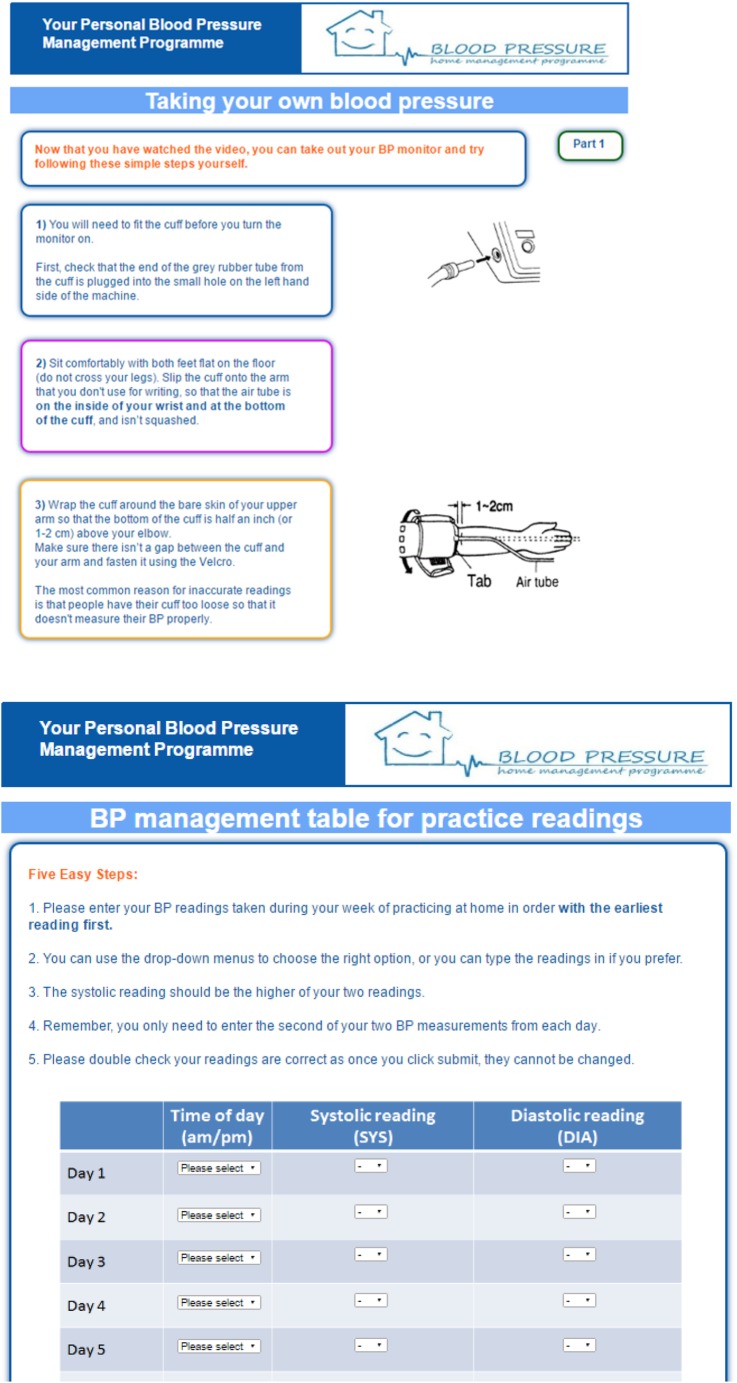
Screenshots of HOME BP pages. HOME BP, Home and Online Management and Evaluation of Blood Pressure.

Patients will be instructed how to correctly undertake self-monitoring to promote patient self-efficacy. An Omron M3 monitor will be given to intervention group participants, and training provided through HOME BP online using a demonstration video. Participants will be asked to rehearse self-monitoring for a minimum of 7 days and enter their practice readings into HOME BP online before they are able to undertake study procedures. Participants will be able to print off tables to record each day's readings off-line and then enter all seven readings at one time (see figure 2 for screenshots of the intervention pages). Patients will also be provided with examples of successful hypertension management using HOME BP procedures to further increase self-efficacy for BP management.

Nine weeks after participants have been allocated to HOME BP, a behaviour change choice tool outlining the specific benefits of user-selected lifestyle modifications targeting healthy eating, physical activity, losing weight and salt and alcohol reduction will be available within HOME BP online. Users will be have the option of reading guidance about the benefits and commitments of each lifestyle change, so that they can choose the one (or more) which would be best for them. Patients will be reassured that additional behavioural support will be provided by their practice HOME BP support provider (usually a nurse or healthcare assistant) for self-monitoring or lifestyle modifications, if required. The rationale for providing these options 9 weeks after the intervention began was to avoid making multiple behaviour changes^39^ until participants had an opportunity to form habits around the key target behaviours of self-monitoring and medication adherence.^40^

#### HOME BP online: healthcare practitioner behavioural components

HOME BP online will build healthcare practitioner motivation, knowledge, skills, positive outcome expectations and self-efficacy for study procedures, particularly medication titration. Healthcare practitioner self-efficacy will be promoted by addressing concerns regarding patient titration acceptance; the reliability of home blood-pressure readings and study procedures as an effective tool for overcoming clinical inertia for patients and practitioners will be outlined. Evidence demonstrating the health-related benefits of the medication titration procedure will be presented, in addition to evidence indicating that the medication escalation procedures do not result in increased patient side effects.^14^ ^20^ ^21^ Users will be provided with information about the study procedures specific to their role (ie, prescriber or support provider) and information relating to study safety procedures.

Patients and healthcare practitioners will be able to re-access their specific behavioural intervention components from HOME BP online at any time throughout the study.

#### Self-monitoring procedures

Patients will be advised to take two morning BP readings for 7 days each month and in each case to use the second reading (as per the TASMINH2 and TASMIN-SR studies).^20^ ^21^ Automated email prompts will be sent to participants as reminders to begin BP self-monitoring. Patients will need to have seven readings to enter into HOME BP online, but will be able to flexibly complete their readings over a 2-week period if required. Following the completion of each set of seven readings, patients will have access to the behavioural intervention components described above, but will not enter further BP readings for further 4 weeks.

#### BP targets

BP targets will be set in line with the National Institute for Health and Care Excellence (NICE) hypertension guidelines,^41^ with adjustment by 5/5 mm Hg for home readings.^42^ People under 80 without diabetes will have BP targets of <135/85 mm Hg, while patients aged 80 and over without diabetes will have a target BP of <145/85 mm Hg. Target BP for all patients with diabetes will be <135/75 mm Hg (those with proteinuria and therefore lower targets are excluded). After the seven readings are entered in to HOME BP online, the mean BP will be calculated and feedback provided to the patient and the healthcare practitioners according to a traffic light system (developed from that used in previous medication titration procedures).^20^ ^21^ These readings will be used to inform the medication titration procedure.

#### Medication titration procedures

All HOME BP prescribers will receive a copy of the NICE hypertension guidelines^41^ and will complete an individualised patient medication review at baseline for intervention group participants, where three potential medication changes will be selected and agreed with the patient. If a patient's mean BP readings are above target for two consecutive months, the medication titration procedure will be initiated by HOME BP online. Patients and practice staff will be informed by email that a medication change is recommended. The prescriber will be asked to implement the preplanned medication change and confirm this with the patient (or explain the reasons for non-implementation, if applicable). If patients decide not to initiate a medication change, the prescriber will receive information provided by the patient outlining the reasons for non-titration. When a recommended medication change is not initiated, an above target reading at the subsequent BP entry will again prompt a medication change recommendation from HOME BP online.

If patients take two BP readings that are too high (>180/110 mm Hg) or low (SBP <100 mm Hg), they will be asked to repeat the measurement after 5 min. If readings are still too high or too low, the HOME BP healthcare practitioners will be alerted by email and patients will be advised to make an appointment with the prescriber in the usual way within 3 days. Decisions about patients' medication will remain at the prescriber's discretion at all times. If a mean target BP (ie, below target but SBP above 100 mm Hg) is entered into HOME BP online for three consecutive months, patients will be advised to reduce BP monitoring to once every 8 weeks as their BP is currently well controlled. This will revert to monthly should subsequent mean BP rise above target.

#### HOME BP behavioural support

Optional additional behavioural support for self-monitoring and lifestyle modifications will be available to all patients via the practice support providers, who will be trained in applying the CARE (Congratulate, Ask, Reassure, Encourage) approach (paper outlining this approach in HOME BP forthcoming). A face-to-face appointment is offered to all participants at 4 weeks following randomisation and 1 week after any lifestyle modifications are introduced, with an option for remote telephone or email support if preferred. A maximum of six face-to-face support sessions will be available if patients experience difficulties associated with self-monitoring or lifestyle change, and patients will have the option to email their support provider through HOME BP online at any point. Template-guided motivating and reassuring support messages will be sent by email from the support provider to all patients every 4 weeks to encourage and reinforce patient adherence to monitoring, medication regimes and lifestyle changes (where applicable).

#### Monitoring adherence

Automated email reminders will be sent to patients and healthcare practitioners at key milestones to promote adherence to study procedures. Adherence to titration procedures will also be monitored by the healthcare practitioners and study team to ensure changes have been enacted (or to understand why not). At the end of the study, each HOME BP supporter will return a copy of their support log. An audit of a selection of BP monitors will be conducted.

### Usual care

Patients allocated to usual care will have access to the information provided in the Blood Pressure UK patient leaflet for hypertension through HOME BP online. They will receive routine hypertension care, with appointments and medication changes made at the GP's discretion.

### Outcomes

The primary outcome of the trial will be difference in office SBP (mm Hg, mean of second/third readings) at 12-month follow-up between the intervention and control groups adjusting for baseline BP, practice, BP target levels and gender. These will be entered and stored on an independent, secure server.

Secondary outcomes (also adjusted for baseline and covariates where appropriate) will comprise:

- Difference in office SBP between intervention and control groups at 6-month follow-up.
- Difference in office DBP between intervention and control groups at 6-month and 12-month follow-up.
- Difference in office SBP and DBP at 6 and 12 months using the mean of the second to sixth BP readings.
- Difference in health-related quality of life measured using EQ-5D-5L.^43^
- Difference in patient enablement measured using the patient enablement instrument.^44^
- Economic modelling (including cost of equipment and drugs, patient and healthcare professional time and support provision).

Process evaluation measures will include:

- Medication beliefs measured using the Beliefs about Medicines Questionnaire (BMQ)^45^ and medication adherence using the Medication Adherence Report Scale (MARS).^46^
- Patient perceived side effects measured using the symptom subscale of the Illness Perception questionnaire (IPQ-R) hypertension.^47^
- Patient self-efficacy.^48^
- Medication use (number and defined daily doses).^49^
- Patient weight measured within clinic and self-reported lifestyle behaviours.
- Prescriber and support provider self-efficacy and outcome expectations.
- Prescriber and support provider confidence in the acceptability of the intervention.

Intervention group only:

- DI website usage and engagement, including lifestyle choice, usage and progress.
- Patient BP monitoring adherence measured by the HOME BP online system.
- Adherence to recommended medication changes.
- Usage of optional support provision.
- Prescriber initiation of titration procedures.

### Adverse events

HOME BP healthcare practitioners will inform investigators of all patient adverse events occurring throughout the trial. The assessment of whether or not an serious adverse event (SAE) is an expected consequence of receiving the intervention will be provided by the chief investigator (or clinical reviewer delegate), it will not be provided by the investigator responsible for the care of the participant.

### Study end points

Participants in the intervention and control groups will be invited to appointments with an independent research nurse for assessment of primary and secondary end points at 6 months and 12 months following entry into the study. These appointments will be conducted in the same way for both groups. Any other contacts with the GP practice (outside of the study) will be recorded in the medical notes and reported for the economic analysis at the end of the trial (based on blind review of patient medical notes). Only members of the study research team involved in the data collection, and data analysis will have access to the final trial data set.

### Measures

See table 1 for full details of study measures.

**Table 1:** Measures and schedule of observations within the HOME BP trial

	Time point				
Measure	Baseline/screening	Visit 1	Visit 2	Intervention group only	Usual care group only
Month	0	6	12	(0–12)	12
Patient sociodemographic measures	X				
Frequency of previous self-monitoring	X				X
Clinical measures					
Systolic BP (SBP)	X	X	X		
Diastolic BP (DBP)	X	X	X		
Weight (kg)	X		X		
Height (cm)	X				
Diabetes status	X				
Hypoglycaemic episodes in last 6 months	X	X	X		
Medication changes (prescriptions issued)			X (NR)		
Consultations			X (NR)		
Patient self-report measures					
Patient enablement instrument	X		X		
Patient self-efficacy	X	X	X		
Beliefs about medication (BMQ)	X	X	X		
Medication adherence (MARS)	X		X		
Patient perceived side effects (IPQ-R hypertension: symptoms subscale)	X		X		
Changes to lifestyle behaviours			X		
Patient objectively recorded measures					
Website usage				X*	
Usage of hypertension pages				X*	
Monitoring of blood pressure					
Self-reported SBP				X*	
Self-reported DBP				X*	
Medication titration recommended				X*	
Titration uptake				X*	
Reasons for non-titration				X*	
Usage of lifestyle pages				X*	
Choice of lifestyle changes				X*	
Reported progress on lifestyle change (eg, weight change)				X*	
HCP objectively recorded measures					
Usage of training pages (prescriber guide)				X	
Emails sent to the patient				X	
Titration procedure compliance				X	
Support provision				X	
HCP self-report measures					
Self-efficacy and outcome expectations	X†				
Confidence in the acceptability of the intervention (for patients)	X†				
Economic measures					
Patient quality of life (EQ-5D)	X	X	X		
Costs of equipment and drugs			X(NR)		
Health professional time			X(NR)		
Patient time			X(NR)		
Qualitative process analysis					
Patient experience and views of the HOME BP				X	X
HCP experience and views of the HOME BP				X	X

*HOME BP intervention arm only—measured throughout the study via HOME BP online.

†Measured directly before and after the HOME BP online training completion.

HOME BP, Home and Online Management and Evaluation of Blood Pressure; NR, notes review.

### Statistical analysis

The principal analysis will use raw and adjusted data and will be performed at the end of the trial after all data have been collected. The primary analysis will use general linear modelling to compare intervention and usual care SBP at follow-up adjusting for baseline BP, practice (as a random effect), BP target levels and gender. Analyses will be on an intention to treat basis with imputation. Secondary analyses will be reported for complete cases. No interim analyses will be performed. A sensitivity analysis will examine the potential effect of missing data using multiple imputation. Planned subgroup analyses will be of BP target groups, older versus younger (65 as threshold), men versus women and better controlled at baseline versus worse controlled at baseline (median SBP). Secondary analyses will use similar techniques to assess differences between groups in SBP at 6 months, DBP at 6 months and 12 months, medication use and quality of life. Additional analyses will examine patient and healthcare professional adherence to advice and study processes, website usage and support provision. Process analyses will be reported elsewhere, but will assess the impact of patient enablement and self-efficacy, symptom perceptions and medication beliefs on outcomes. These analyses will also examine the relationship between support uptake, engagement with healthy behaviour change and outcomes.

### Economic analysis

TASMINH2^20^ resulted in a 5.4 mm Hg reduction in BP in self-management compared with usual care which was cost-effective with £1.6k per QALY for men and £4.9k for women. Thus if HOME BP results in the hypothesised difference of 5 mm Hg, then using the same cost-effectiveness model, it will be highly cost-effective—unless of course the costs are much greater. The intervention cost in TASMINH2 was £105, which included the BP monitor, tele-monitoring connection and training costs (see table 2 in Kaambwa *et al*^50^). Key differences between the interventions in HOME BP and TASMINH2 have to do with the use of behavioural techniques in the former, including guidance about lifestyle change and support in making such changes as well as recommendations to change medications with recording of whether such changes were made and further reminders if not. HOME BP thus tests the intervention as delivered digitally and the additional value of behavioural support in self-management. To facilitate costing of the behavioural add-ons to self-management, the costing of the intervention in HOME BP as far as possible will separate these elements.

#### Economic analysis plan

A within trial analysis will estimate cost per unit reduction in SBP, the primary outcome, with adjustments in that outcome as described in the ‘Statistical analysis’ section, including subgroups. Costs will include those due to the intervention, as well as those due to changes in medication, use of other relevant NHS resources and any costs borne by patients such as in diet and lifestyle. NHS and societal costs will be estimated and uncertainty explored in sensitivity analyses. To facilitate analysis of the difference between the intervention in HOME BP and that of self-management, the components and the cost of the intervention in HOME BP will be compared with that of TASMINH2.

These short-term results will provide input to a long-term cost-effectiveness model, based on that developed for TASMINH2, updated by subsequent work as relevant. Since these studies all show reductions in BP as cost reducing or highly cost-effective, this modelling will address the cost-effectiveness of the HOME BP-intervention versus usual care but also of the addition of the behavioural/digital element relative to paper-based self-management without that element. The HOME BP trial will collect data on numbers and types of antihypertensive prescriptions between arms.

### Qualitative substudy

The qualitative interviews will seek to provide an in-depth understanding of the factors that may facilitate or diminish the acceptability of the HOME BP intervention, and adherence to implementation from the perspective of patients and healthcare professionals using HOME BP. Interviews and focus groups will be undertaken alongside participation and after participants finish the HOME BP intervention for patients in the intervention and usual care groups. Purposive sampling will be used to select patients from the intervention group by age, gender, socioeconomic status and level of engagement with the website to allow for a wide range of views and experiences of the HOME BP online and the HOME BP intervention. Open-ended inductive questions will be used to elicit user perspectives and experiences of the intervention and the support provided, allowing participants to freely describe their experiences and views in their own way and to focus on whatever is most salient to them. Focus group discussions and interviews will also be conducted with healthcare professionals who have been involved in the trial procedures or intervention delivery. Interviews and focus groups will be audio-recorded, fully transcribed and analysed thematically.

## Ethics and dissemination

### Ethical approval

Ethical approval for the HOME BP study has been obtained from the South Central—Hampshire A Ethics Committee (reference: 15/SC/0082). R&D approvals have been obtained from all relevant Clinical Research Networks. All substantial amendments must be approved by the University Ethics Committee and NHS Ethics Committee responsible for the trial, in addition to approval by NHS R&D. Investigators are kept up to date with relevant changes via regular management group meetings.

### Data monitoring

The Programme Steering Committee is responsible for ensuring programme adherence to the protocol, and adherence to the requirements of the Guidelines for Good Clinical Practice. It was decided by the Steering Committee that a data monitoring committee was not required. The trial may be subject to inspection and audit by University of Southampton, under their remit as sponsor, the trial coordinating centre as the Sponsor's delegate and other regulatory bodies.

### Dissemination

Dissemination of our research will be via multiple pathways. We will submit the primary, secondary and tertiary study results for publication in highly cited and open access peer-reviewed journals and present findings at national and international conferences. We will also provide summaries of HOME BP findings to professional societies (such as British Hypertension Society), patient groups (Blood Pressure UK), participants, NHS organisations and healthcare providers. We will also disseminate to the public through regular press releases and to stakeholders through interactive workshops.

If cost-effective, the HOME BP intervention will be disseminated for clinical use by University of Southampton or a suitable, appropriate licensed partner. Following the Behavioral Intervention Technology model,^51^ we anticipate that future technology enhancements in mode of delivery of the intervention will be possible and desirable without the need to re-evaluate the essential behaviour support content of the intervention. For example, as smartphone use becomes more prevalent in the target population it will be easy to transfer the content of HOME BP for delivery by smartphone, with automatic transfer of BP readings (which will reduce the potential for patient error).
